# The influence of the COVID-19 pandemic on measles vaccination coverage

**DOI:** 10.1590/0102-311XEN183924

**Published:** 2025-07-18

**Authors:** Gustavo de Almeida Santos, Expedito José de Albuquerque Luna

**Affiliations:** 1 Faculdade de Medicina, Universidade de São Paulo, São Paulo, Brasil.

**Keywords:** COVID-19 Pandemic, Vaccination, Immunization, Measles Vaccine, Systematic Review, Pandemia por COVID-19, Vacinação, Imunização, Vacina contra Sarampo, Revisão Sistemática, Pandemia de COVID-19, Vacunación, Inmunización, Vacuna Antisarampión, Revisión Sistemática

## Abstract

We conducted a systematic review to assess the impact of the COVID-19 pandemic on measles vaccination coverage. We searched for articles published between January 2021 and December 2023 in Portuguese, English, and Spanish in the Web of Science, ScienceDirect, PubMed, and LILACS databases. The final sample consisted of 32 studies, which demonstrated that most countries had a 1% to 10% decrease on measles vaccination coverage during the pandemic. However, the influence of the pandemic varied worldwide, ranging from 1% to 60% based on the region. The COVID-19 pandemic has had a relatively modest impact on measles immunization, with a complex intersection of several factors associated with the decrease in measles vaccination coverage.

## Resumo

Realizamos uma revisão sistemática para avaliar o impacto da pandemia de COVID-19 na cobertura vacinal contra o sarampo. Artigos publicados entre janeiro de 2021 e dezembro de 2023 em português, inglês e espanhol nas bases de dados Web of Science, ScienceDirect, PubMed e LILACS foram pesquisados. A amostra final incluiu 32 estudos que mostraram que a maioria dos países sofreu uma redução de 1% a 10% na cobertura vacinal contra o sarampo durante a pandemia. No entanto, a influência da pandemia variou em todo o mundo (de 1% a 60 % dependendo da região). A pandemia de COVID-19 teve um impacto relativamente modesto na imunização contra o sarampo, mostrando uma interseção complexa de vários fatores associados à diminuição da cobertura vacinal contra o sarampo.

## Resumen

Se realizó una revisión sistemática para evaluar el impacto de la pandemia de COVID-19 en la cobertura de vacunación contra el sarampión. Se realizaron búsquedas de artículos publicados entre enero de 2021 y diciembre de 2023 en portugués, inglés y español en las bases de datos Web of Science, ScienceDirect, PubMed y LILACS. La muestra final constó de 32 estudios, que demostraron que la mayoría de los países tuvieron una disminución del 1% al 10% en la cobertura de vacunación contra el sarampión durante la pandemia. Sin embargo, la influencia de la pandemia varió a nivel mundial, oscilando entre el 1% y el 60% según la región. La pandemia de COVID-19 ha tenido un impacto relativamente modesto en la inmunización contra el sarampión, con una intersección compleja de varios factores asociados con la disminución de la cobertura de vacunación contra el sarampión.

## Introduction

COVID-19 is a viral disease that considerably impacted the world, with more than 760 million cases and 6.9 million deaths recorded since December 2019. However, these figures could be even higher, considering possible underreporting and undiagnosed cases ^1^. The COVID-19 pandemic has had heterogeneous effects on different populations, varying according to factors such as age and socioeconomic status. This scenario led children and adolescents to face particular challenges, influenced not only by their stage of development, but also by the way the disease and its associated control strategies impacted their lives ^2^. According to data from the World Health Organization’s (WHO) Pulse survey on the continuity of essential health services during the pandemic, most countries (90%) have reported interruptions in essential health services since the health crisis began. These disruptions were especially notable in critical areas for child welfare, such as routine immunization, affecting around 70% of outreach services and 61% of facility-based services ^3^.

Measles, among vaccine-preventable diseases, is a major threat to public health, characterized by its severity and very high transmission potential. Caused by a virus, this illness can result in serious complications and even death. The effective implementation of measles vaccination has been crucial in reducing the morbidity and mortality associated with the disease. From 2000 to 2022, vaccination prevented approximately 57 million measles-related deaths. Despite the historical progress of measles vaccination coverage over the past decades, concerning data suggest that, recently, this progress has stagnated and even regressed. In 2016, for example, the Americas lost its certification as a measles-free region due to outbreaks in Venezuela and Brazil ^4^. In 2022, only around 83% of children worldwide received a measles vaccine dose during their first year of life by routine health services - the lowest rate since 2008. This scenario is concerning, especially considering that measles vaccine is widely available, safe, and affordable. Despite vaccine availability, statistics reveal a considerable number of measles-related deaths in 2022, totaling around 136,000 deaths globally and reflecting a worrying reality, especially among unvaccinated or under-vaccinated children up to five years old ^5^.

According to the WHO, the COVID-19 pandemic caused setbacks in global immunization and surveillance efforts, leaving millions of children vulnerable to preventable diseases ^5^. As aforementioned, immunization against measles is a highly effective public health intervention in preventing morbidities and mortality associated with this disease. However, the pandemic has also triggered a series of unprecedented challenges for health systems worldwide. Control measures adopted to contain the spread of the new coronavirus, such as social distancing, lockdowns, and mobility restrictions, have significantly affected health services, including immunization programs ^3^ ^,^ ^6^ ^,^ ^7^.

Given this context, there is a pressing need to better understand the impact of the COVID-19 pandemic on measles immunization, since concerns have been raised about possible setbacks in advances made in measles prevention. Additionally, the coexistence of the pandemic with measles outbreaks could further exacerbate negative consequences for public health. Therefore, this systematic review aims to fill this knowledge gap by providing a comprehensive, evidence-based analysis of the impact of the COVID-19 pandemic on measles immunization. Understanding the dimensions of this impact is fundamental to guide public health policies and strategies focused on mitigating the adverse effects of the pandemic on the prevention of vaccine-preventable diseases such as measles. This scientific research can inform effective and targeted interventions aimed at ensuring the continuity of immunization programs and protecting the health of vulnerable populations in the midst of the COVID-19 pandemic.

## Methods

### Literature review protocol

This is a literature systematic review, whose protocol was previously registered with the Prospective International Registry of Systematic Reviews (PROSPERO; registration n. CRD42024498943). This review adhered to the *Preferred Reporting Items for Systematic Reviews and Meta-Analyses* (PRISMA) guidelines ^8^ in order to ensure the scientific rigor inherent in the process. To assess the quality/risk of bias of the included studies, the *Effective Public Health Practice Project Quality - Assessment Tool for Quantitative Studies* (https://merst.healthsci.mcmaster.ca/ephpp/) was used. Box 1 shows the results.

**Box 1:** Quality assessment/risk of bias of included studies according to the *Effective Public Health Practice Project - Quality Assessment Tool for Quantitative Studies*.

STUDY	SELECTION BIAS	STUDY DESIGN	CONFOUNDERS	BLINDING	DATA COLLECTION METHODS	WITHDRAWALS AND DROP-OUTS	GLOBAL RATING
Abid et al. ^9^	Strong	Weak	Strong	Strong	Strong	N/A	Moderate
Abu-rish et al. ^10^	Strong	Weak	Strong	Strong	Strong	N/A	Moderate
Ackerson et al. ^11^	Strong	Moderate	Strong	Strong	Strong	N/A	Strong
Aizawa et al. ^12^	Strong	Weak	Strong	Strong	Strong	N/A	Moderate
Alhaddad et al. ^13^	Strong	Weak	Strong	Strong	Strong	N/A	Moderate
Babatunde et al. ^14^	Weak	Weak	Weak	Strong	Strong	N/A	Weak
Buck et al. ^15^	Strong	Moderate	Strong	Strong	Strong	N/A	Strong
da Silva et al. ^16^	Strong	Weak	Strong	Strong	Strong	N/A	Moderate
Eiden et al. ^17^	Strong	Weak	Strong	Strong	Strong	N/A	Moderate
Elmi et al. ^18^	Strong	Weak	Strong	Strong	Strong	N/A	Moderate
Firman et al. ^19^	Strong	Moderate	Strong	Strong	Strong	N/A	Strong
Herdea et al. ^20^	Strong	Moderate	Strong	Strong	Strong	N/A	Strong
Kiely et al. ^21^	Strong	Weak	Strong	Strong	Strong	N/A	Moderate
Lai et al. ^22^	Weak	Weak	Weak	Strong	Strong	N/A	Weak
Lucinde et al. ^23^	Strong	Moderate	Strong	Strong	Strong	N/A	Strong
MacDonald et al. ^24^	Strong	Moderate	Strong	Strong	Strong	N/A	Strong
Masnour et al. ^25^	Weak	Weak	Strong	Strong	Strong	N/A	Weak
Martínez-Marcos et al. ^26^	Strong	Weak	Strong	Strong	Strong	N/A	Moderate
McQuaid et al. ^27^	Strong	Weak	Strong	Strong	Strong	N/A	Moderate
Middeldorp et al. ^28^	Moderate	Weak	Strong	Strong	Strong	N/A	Moderate
Nuzhath et al. ^29^	Strong	Moderate	Strong	Strong	Strong	N/A	Moderate
Osei et al. ^30^	Strong	Moderate	Strong	Strong	Strong	N/A	Moderate
Procianoy et al. ^31^	Strong	Weak	Strong	Strong	Strong	N/A	Moderate
Rahman et al. ^32^	Strong	Moderate	Strong	Strong	Strong	N/A	Strong
Sato et al. ^33^	Strong	Weak	Strong	Strong	Strong	N/A	Moderate
Shet et al. ^34^	Strong	Weak	Strong	Strong	Strong	N/A	Moderate
Summan et al. ^35^	Strong	Moderate	Strong	Strong	Strong	N/A	Strong
Taine et al. ^36^	Strong	Moderate	Strong	Strong	Strong	N/A	Strong
Thsehla et al. ^37^	Strong	Moderate	Strong	Strong	Strong	N/A	Strong
Ullah et al. ^38^	Strong	Weak	Strong	Strong	Strong	N/A	Moderate
Zhong et al. ^39^	Strong	Moderate	Strong	Strong	Strong	N/A	Strong
Zürcher et al. ^40^	Strong	Moderate	Strong	Strong	Strong	N/A	Strong

N/A: not applicable.

Note: the global rating of the Effective Public Health Practice Project (EPHPP) assesses the methodological quality of included studies based on three categories: “strong”, “moderate”, and “weak”. This classification considers methodological components such as sample selection, bias, data collection, and analysis. Studies classified as “strong” are those that do not present any component rated as “weak”. Studies with a “moderate” rating have exactly one component classified as “weak”, indicating a specific methodological limitation that does not significantly compromise the overall quality of the study. Finally, studies classified as “weak” have two or more components rated as “weak”, reflecting greater vulnerability in the validity and reliability of the results. This approach provides a thorough and transparent assessment of the quality of the studies, aiding in the interpretation of findings and identifying potential limitations.

### Data sources and research strategies

Initially, the search period was set to include articles published from January 2020 to December 2023, based on the beginning of the COVID-19 pandemic in 2020. However, during the review development, it was decided to restrict the search to articles published from January 2021 to December 2023. The exclusion of articles published in 2020 is due to, during this period, health systems responses and impacts on vaccination campaigns were still in early adaptation stages and did not yet fully represent the global consequences of the pandemic. The period from 2021 to 2023 guarantees the inclusion of data in a context in which policies to tackle COVID-19 were already consolidated and the impacts of vaccination campaigns, including measles, were fully manifested. This interval makes it possible to accurately observe the prolonged effects of the pandemic on vaccination coverage, reflecting a more stable and representative scenario of changes within health systems.

**Figure 1:**
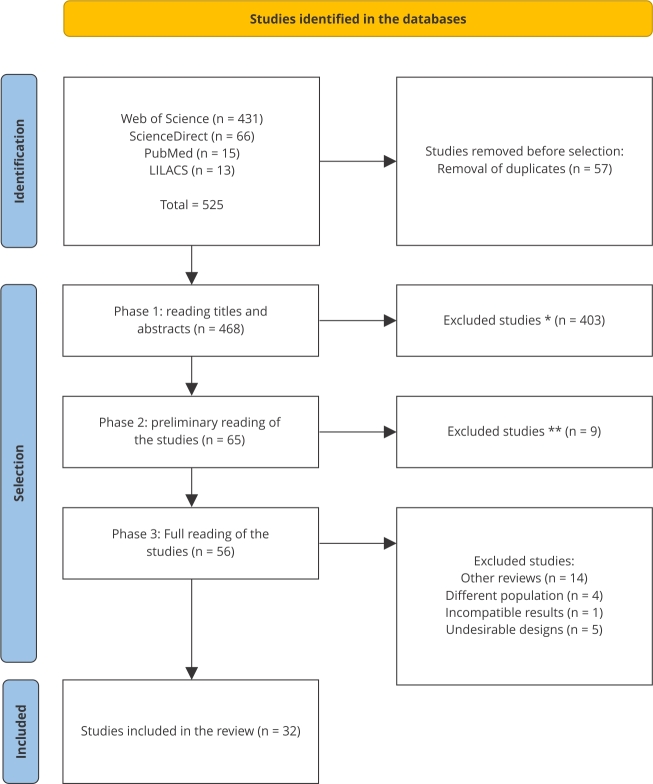
Flowchart of the research conducted according to the *Preferred Reporting Items for Systematic Reviews and Meta-Analysis* (PRISMA).

Search strategies were conducted in a structured way in the Web of Science, ScienceDirect, PubMed, and LILACS databases. Searches took place between December 20 and 22, 2023. The search string used in each database was adapted according to the specific syntax of each one, ensuring the inclusion of terms relevant to the review. The following descriptors were used, previously selected by Health Science Descriptors (https://decs.bvsalud.org/en/): “(Sarampo OR Measles OR Sarampión) AND (Covid-19 OR Covid 19)”. To ensure reproducibility, the search only considered human studies, with no restrictions on language or year of publication, and filters such as period of publication of the articles, language, and type of work were applied. Figure 1 summarizes the selection process.

### Inclusion and exclusion criteria

For this review, cross-sectional studies, retrospective cohorts, interrupted time series and other ecological studies published in Portuguese, English, or Spanish from 2021 to 2023, investigating the impact of the COVID-19 pandemic on measles vaccination coverage were included. Relevant research that explored causes of immunization variation, such as socioeconomic factors, health policies, access to services, and changes in vaccination programs were considered. Only peer-reviewed studies were included to guarantee data quality. Studies with incomplete data, duplicates, publications in languages other than Portuguese, English and Spanish , non-human studies, other reviews on the subject, and exclusive predictive modeling on the impact of COVID-19 on vaccination coverage were excluded. A comprehensive search of the references of similar reviews was also conducted to identify additional articles that met the inclusion criteria.

### Selection process

After defining the descriptors, the LILACS, Science Direct, Web of Science, and PubMed databases were searched, generating RIS and NBIB files. Integrated with EndNote Web (https://web.endnote.com) and Rayyan (https://www.rayyan.ai/), duplicates were removed using semi-automatic tools. Duplicates confirmed by the authors were identified and excluded. Afterwards, a three-stage selection process was conducted: reviewing titles and abstracts, preliminary eligibility reading, and detailed reading of the selected studies. Two independent reviewers assessed the papers, with a third consulted in case of disagreement (Figure 1).

* Studies were excluded because they were not aligned with the scope and specific objectives of this systematic review, which focuses on the impact of the COVID-19 pandemic on measles immunization/coverage. The decision was therefore made to limit the analysis to works that directly contribute to the understanding and discussion of the topics covered, guaranteeing the cohesion and relevance of the study; ** Studies were excluded due to incomplete and insufficient data to meet the inclusion criteria established. This decision was made to ensure the integrity and robustness of the analysis, prioritizing the inclusion of studies that present relevant and substantial information for the proposed research.

### Data extraction

After selection, the articles were organized in spreadsheets using Microsoft Excel (https://products.office.com/). Authors conducted a thorough reading to extract relevant information, categorizing it into three groups: (1) “Characterization of the included studies”, including authors, journal, country of origin, objectives, and methods; (2) “Relevant information”, such as sample size, results, conclusions, and additional observations; and (3) “Answer to the research question”, focused on the impact of the COVID-19 pandemic on measles immunization, exploring challenges and solutions to improve vaccination coverage during this period. This approach ensured a comprehensive and structured data analysis, accounting for all relevant information to answer the research questions. The categorization of data into three groups constituted a methodological strategy to organize, analyze, and systematically present information collected in this review. This approach was essential to manage data volume, ensuring they were grouped into logical blocks aligned with the study’s main objectives. This enabled the performance of descriptive analyses, facilitating the identification of trends and patterns. Furthermore, structuring data into specific categories contributed to clearer result communication, making the presented information easier to understand and interpret.

The data were extracted in pairs and independently by two reviewers. Each reviewer extracted the data independently and, then, extractions were compared. Any disagreements were resolved by discussion between the reviewers or with a third reviewer’s intervention when necessary, ensuring greater reliability in the extraction process.

### Data analysis

To interpret and present the results, descriptive statistics were used to structure, summarize, and detail essential aspects of the main variables analyzed. When comparing the data, the predominant trends found in the results were outlined, highlighting similarities, discrepancies, and particularities, as addressed in each study.

Regarding data interpretation, Category 1 shows the most frequent data, using the GraphPad Prism tool, version 8.0.2 (https://www.graphpad.com/), to quantify the absolute number and calculate the proportion in relation to the total to characterize the studies. In Category 2, the most frequent results were explored, following the approach of Category 1, but also singular results, as long as they were important. Category 3 covers all results, whether they agree or not. Thus, unlike Categories 1 and 2, in which presenting the most frequently reported results was a priority, Category 3 aimed to integrate and discuss the findings regardless of whether they were concordant or discordant, highlighting both the identified patterns and potential exceptions. This approach enabled a more comprehensive and representative analysis of the data diversity presented by the included studies.

## Results

### Characterization of the sample studies

The final sample consisted of 32 studies (Box 2) ^9^ ^,^ ^10^ ^,^ ^11^ ^,^ ^12^ ^,^ ^13^ ^,^ ^14^ ^,^ ^15^ ^,^ ^16^ ^,^ ^17^ ^,^ ^18^ ^,^ ^19^ ^,^ ^20^ ^,^ ^21^ ^,^ ^22^ ^,^ ^23^ ^,^ ^24^ ^,^ ^25^ ^,^ ^26^ ^,^ ^27^ ^,^ ^28^ ^,^ ^29^ ^,^ ^30^ ^,^ ^31^ ^,^ ^32^ ^,^ ^33^ ^,^ ^34^ ^,^ ^35^ ^,^ ^36^ ^,^ ^37^ ^,^ ^38^ ^,^ ^39^ ^,^ ^40^. These articles were published in 22 wide-ranging scientific journals, particularly the *Vaccine*, which emerged as the most prominent in its approach to the subject under investigation. Regarding quality of the included studies, the analysis conducted using the *Effective Public Health Practice Project - Quality Assessment Tool for Quantitative Studies*, 37% of the articles were classified as “strong” and 53% as “moderate”, as seen in Box 1.

**Box 2:** Characteristics of studies on the impact of COVID-19 on measles immunization.

STUDY	JOURNAL	COUNTRY	LANGUAGE	OBJECTIVE	STUDY TYPE
Abid et al. ^9^	*Risk Management and Healthcare Policy*	Afghanistan	English	Direct impact of the pandemic on vaccination coverage and child immunization	Cross-sectional
Abu-rish et al. ^10^	*International Journal of Clinical Practice*	Jordan	English	Direct impact of the pandemic on vaccination coverage and child immunization	Cross-sectional
Ackerson et al. ^11^	*Pediatrics*	United States	English	Direct impact of the pandemic on vaccination coverage and child immunization	Retrospective cohort
Aizawa et al. ^12^	*Vaccine*	Japan	English	Direct impact of the pandemic on vaccination coverage and child immunization	Ecological
Alhaddad et al. ^13^	*Epidemiology and Health*	Iraq	English	Direct impact of the pandemic on vaccination coverage and child immunization	Cross-sectional
Babatunde et al. ^14^	*Pan African Medical Journal*	Nigeria	English	Direct impact of the pandemic on vaccination coverage and child immunization	Interrupted time series
Buck et al. ^15^	*Public Health*	United Kingdom	English	Direct impact of the pandemic on vaccination coverage and child immunization	Cross-sectional
da Silva et al. ^16^	*BMC Infectious Disease*	Brazil	English	Analysis of changes in vaccine administration and coverage during the pandemic	Ecological
Eiden et al. ^17^	*Expert Review of Vaccines*	United States	English	Analysis of changes in vaccine administration and coverage during the pandemic	Cross-sectional
Elmi et al. ^18^	*Journal of Tropical Pediatrics*	South Africa	English	Direct impact of the pandemic on vaccination coverage and child immunization	Cross-sectional
Firman et al. ^19^	*BJM Open*	United Kingdom	English	Analysis of determinants and trends in vaccination coverage during the pandemic	Retrospective cohort
Herdea et al. ^20^	*Children (Basel)*	Romania	English	Analysis of determinants and trends in vaccination coverage during the pandemic	Retrospective cohort
Kiely et al. ^21^	*Human Vaccines & Immunotherapeutics*	Canada	English	Direct impact of the pandemic on vaccination coverage and child immunization	Ecological
Lai et al. ^22^	*eClinicalMedicine*	Global	English	Analysis of determinants and trends in vaccination coverage during the pandemic	Ecological
Lucinde et al. ^23^	*Vaccine*	Kenya	English	Analysis of determinants and trends in vaccination coverage during the pandemic	Retrospective cohort
MacDonald et al. ^24^	*BMJ Open*	Canada	English	Direct impact of the pandemic on vaccination coverage and child immunization	Retrospective cohort
Masnour et al. ^25^	*PLoS One*	Lebanon and Middle East and North Africa region	English	Analysis of determinants and trends in vaccination coverage during the pandemic	Cross-sectional
Martínez-Marcos et al. ^26^	*Public Health*	Spain	English	Direct impact of the pandemic on vaccination coverage and child immunization	Ecological
McQuaid et al. ^27^	*PLoS Medicine*	Scotland and England	English	Analysis of changes in vaccine administration and coverage during the pandemic	Ecological
Middeldorp et al. ^28^	*Vaccine*	Netherlands	English	Direct impact of the pandemic on vaccination coverage and child immunization	Retrospective cohort
Nuzhath et al. ^29^	*Vaccine*	United States	English	Analysis of determinants and trends in vaccination coverage during the pandemic	Retrospective cohort
Osei et al. ^30^	*Vaccine*	Gambia	English	Direct impact of the pandemic on vaccination coverage and child immunization	Interrupted time series
Procianoy et al. ^31^	*Ciência & Saúde Coletiva*	Brazil	Portuguese	Direct impact of the pandemic on vaccination coverage and child immunization	Ecological
Rahman et al. ^32^	*Human Vaccines & Immunotherapeutics*	Pakistan	English	Direct impact of the pandemic on vaccination coverage and child immunization	Retrospective cohort
Sato et al. ^33^	*Ciência & Saúde Coletiva*	Brazil	Portuguese	Analysis of determinants and trends in vaccination coverage during the pandemic	Ecological
Shet et al. ^34^	*Lancet Global Health*	Global	English	Direct impact of the pandemic on vaccination coverage and child immunization	Ecological
Summan et al. ^35^	*The Lancet Regional Health. Southeast Asia*	India	English	Direct impact of the pandemic on vaccination coverage and child immunization	Retrospective cohort
Taine et al. ^36^	*Frontiers in Pediatrics*	France	English	Analysis of changes in vaccine administration and coverage during the pandemic	Interrupted time series
Thsehla et al. ^37^	*Global Health Action*	South Africa	English	Direct impact of the pandemic on vaccination coverage and child immunization	Interrupted time series
Ullah et al. ^38^	*Avicenna*	Pakistan	English	Direct impact of the pandemic on vaccination coverage and child immunization	Cross-sectional
Zhong et al. ^39^	*Vaccine*	Singapore	English	Analysis of changes in vaccine administration and coverage during the pandemic	Retrospective cohort
Zürcher et al. ^40^	*Vaccine*	Switzerland	English	Direct impact of the pandemic on vaccination coverage and child immunization	Retrospective cohort

Among countries that have investigated the influence of COVID-19 on primary health care, focusing on immunizing children, studies were identified in 23 different nations. Brazil and the United States stand out, each with three studies. After expanding the analysis to the six regions defined by the WHO for reporting, analysis, and management purposes, we observed that Europe and the Americas have contributed with most studies on the subject.

As for the objectives of the studies, they were classified into three categories: (1) Direct impact of the pandemic on vaccination coverage and child immunization; (2) Analysis of changes in vaccine administration and coverage during the pandemic; and (3) Analysis of determinants and trends in vaccination coverage during the pandemic. Most studies (62.5%) focused on investigating the direct impact of the pandemic on vaccination coverage and child immunization, as shown in Box 2.

Studies adopting a retrospective cohort methodology were the most frequent (34%), followed by ecological studies (28%) and cross-sectional studies (25%). As for types of data used, most used secondary data (88%), although some studies combined primary and secondary data.

### Sample details

**Figure 2:**
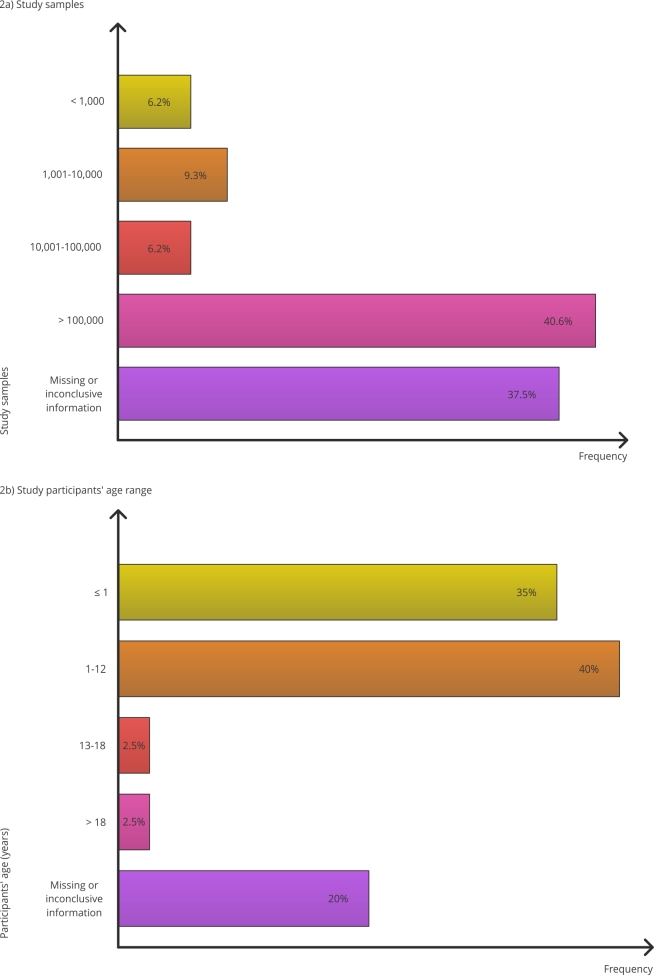
Study samples and age range of study participants.

When analyzing the samples used by the studies included, it was found that most (40.6%) used a sample that exceeded 100,000 individuals. Regarding age range, most studies focused on children aged one to 12 years (40%); however, it is important to note that 35% of the studies addressed children aged one year or younger. Additionally, some studies covered multiple age groups, resulting in the denominator for the age group category (n = 40) exceeding the total number of studies (n = 32). Figure 2 shows these data.

In this study, we chose to consider information from at least half of the included studies. However, it is important to note that certain data, such as information on gender and authors’ suggestions and observations did not reach the minimum number required to carry out a comprehensive descriptive analysis. Therefore, despite their possible relevance, these aspects could not be adequately assessed in this review. Since we aimed to ensure the results were representative and robust, we sought to avoid information from a limited number of studies compromising the representativeness of the overall sample in the review, which could lead to erroneous interpretations. This methodological criterion aims to ensure that conclusions are based on consistent and widely representative data, in line with the study’s objectives and the need for reliable analysis.

### Impact of the COVID-19 pandemic on measles immunization

**Figure 3:**
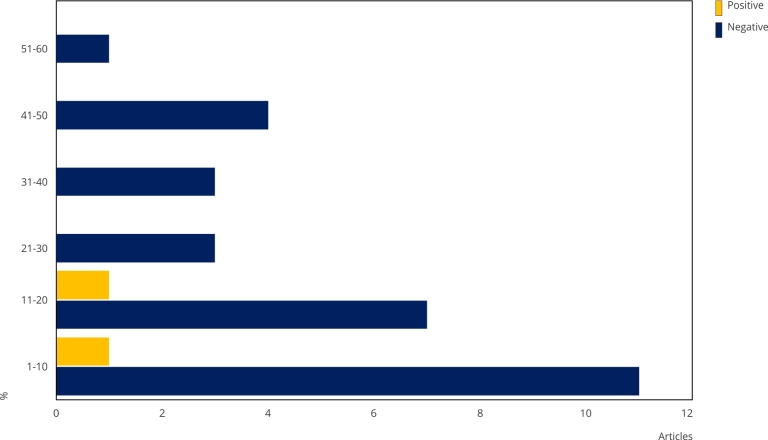
Variation in measles vaccination coverage after the COVID-19 pandemic according to the articles.

Based on our findings, the influence of the COVID-19 pandemic on measles immunization varied substantially between regions, resulting in a 1% to 60% drop in vaccination coverage in the countries studied. This decrease was particularly noticeable during lockdowns, during the acute phase of the pandemic, with varied lengths across different locations. Overall, we found a 1% to 10% reduction in measles immunization during the pandemic in several of the nations analyzed, as shown in Figure 3. After the end of lockdowns, some countries were able to quickly recover vaccination rates, while others faced major challenges in restoring primary health services, especially child vaccination. European studies have indicated intensified child immunization services during the pandemic, aiming to mitigate other health issues related to vaccine-preventable diseases. This was due to a downward trend in vaccination coverage among both children and adults in years prior to the pandemic. These studies documented a 1% to 20% increase in measles immunization during the pandemic, reflecting efforts to address public health challenges arising from the interruption of vaccination services during this critical period.

Regarding reduction in vaccination coverage observed in the studies analyzed, among the WHO Member States, more studies were identified in the Americas. In these, a 10% to 20% decrease in vaccination coverage has predominantly been documented, although some studies have reported more substantial drops, reaching up to 41%-50%. Studies conducted in Brazil show similar outcomes. Europe was second in terms of number of studies identified, with most registering a 1% to 10% reduction in vaccination coverage. This suggests a less pronounced trend in the impact of the COVID-19 pandemic on measles immunization compared to that observed in the Americas.

Some studies explored factors associated with the reduction in vaccination coverage during the pandemic, although few directly addressed underlying causes. These factors are complex and interconnected, varying according to the context of each country. In Afghanistan, for example, the vaccination coverage decrease was attributed to reduced healthcare delivery, insecurity, disruptions in global production and supply chains, border closures, and the temporary suspension of manufacturing vaccines other than COVID-19 by companies. This resulted in shortages of vaccines and other essential supplies. Additionally, fear of COVID-19 exposure and high-risk messaging led people to avoid leaving their homes and seeking routine healthcare. Such insecurity, as well as frequent armed conflicts, further limited access to healthcare ^9^. In Jordan, the largest decline was observed during lockdown, followed by an additional impact with the first wave of COVID-19 ^10^. In the United Kingdom and Brazil, social distancing and isolation, respectively, were highlighted as possible causes for the reduction in vaccine administration, such as measles, mumps, and rubella (MMR) vaccine ^15^ ^,^ ^16^. In Romania, fear of vaccine side effects, antivaccination campaigns in the media, and the spread of false information (infodemic) negatively influenced parents’ decisions to vaccinate their children ^20^. In Canada, the redeployment of healthcare professionals to respond to the pandemic, the use of teleconsultations, and parents’ concerns about visiting healthcare facilities with their children impacted vaccination among children under two years old ^21^. In Singapore, parental hesitancy to visit healthcare facilities due to fear of COVID-19 transmission and logistical challenges posed by strict social distancing measures were major causes for the reduction in vaccination coverage ^39^.

## Discussion

### Fall and regional variation in the influence of the pandemic on measles immunization

Considering our main results, one noteworthy observation was the regional variation in the influence of the COVID-19 pandemic on measles vaccination coverage, resulting in significant decreases that varied considerably across the nations analyzed. A survey conducted by the Kaiser Permanente Southern California (KPSC) health system in California (United States) ^11^, revealed that administration of vaccines against measles in children under 24 months old decreased by 60% in the week of March 22, 2020 compared to the same period in 2019. Although these rates subsequently increased, they remained below the weekly administration rates observed in 2019 until the end of the research period. A study conducted in South Africa ^37^ showed a 2.4% decrease in the application of the first dose of the measles vaccine and 6.9% in full immunization. This demonstrates the wide variation found in our study.

According to data from the WHO, up to the time of this research, Europe was the region affected the most by the COVID-19 pandemic, with a cumulative incidence of approximately 30%, followed by the Americas, with 193 million cases and a cumulative incidence of almost 20% ^41^. Although the Americas rank third in terms of number of COVID-19 cases reported to the WHO, its countries have had the most difficulties in terms of measles immunization during the pandemic. Meanwhile, Europe, which was most impacted by the pandemic, was among the least affected in terms of measles vaccination coverage. This diversity in the impact of the COVID-19 pandemic on countries and regions may be associated with the wide range of variations observed in the influence of the pandemic on measles vaccination coverage.

A global study part of the 2020 *Global Burden of Diseases, Injuries, and Risk Factors Study* (GBD), showed that, from 2010 to 2019, the progression in vaccination coverage of DTP3, MCV1, and Pol3 stagnated or regressed in several locations ^42^. This indicates that, even before the pandemic, there was already a global downward trend in measles immunization. One of the main factors contributing to this drop in recent years is the anti-vaccine movement, driven by misperceptions about vaccine risks ^43^. The evaluation of unfavorable attitudes towards vaccines should be a priority on the global scientific agenda, as these anti-vaccine contingents are detrimental to mass immunization, causing drops in vaccination coverage and raising doubts about vaccine safety and efficacy among certain groups ^44^.

Similarly, regarding adherence to vaccination in the United States, some authors have observed a significant change of at least 30% in the proportion of individuals willing to be vaccinated as the risk of infection increases ^45^. Moreover, the authors identified that risk of mortality generates a greater willingness to vaccination compared to morbidity. They also found that older people were more willing to get vaccinated than younger people, and revealed that individuals with higher income (above USD 90,000) are more willing to get vaccinated than those with lower income. Furthermore, men are more likely to be vaccinated than women, and willingness to be vaccinated can vary according to political ideology and the level of perceived risk ^45^.

The pandemic has brought significant challenges to health systems and communities worldwide, highlighting structural weaknesses and underscoring the interconnections between the health, social, and economic spheres ^46^. It also exposed disparities in access to health services and their impact on the quality of care. The concept of health inequalities requires a comprehensive reformulation to address the extent of disadvantage and discrepancies in the provision of care and health outcomes ^47^. This indicates the urgent need to redistribute resources, funding, labor, and services to promote effective equity in health systems.

According to the analysis conducted, it is clear there is no singular explanation for the declines observed in measles vaccination coverage rates in various nations, nor is there uniformity in the patterns of these reductions across different locations. Instead, we understand that a complex intersection of several factors is associated with the decrease in vaccination coverage against measles and possibly other vaccines during the COVID-19 pandemic. These factors may include, but are not limited to, inertia or even decline in adherence to immunization practices, preexisting structural deficiencies in health systems whose weaknesses have been exacerbated during this critical period, unequal access to health services, previous reductions in vaccination coverage prior to the pandemic, influence of anti-vaccine movements, and even varied intensity of the impact of COVID-19 across different regions. This analysis reflects the multifaceted complexity that permeates immunization dynamics during the pandemic, highlighting the need for a holistic approach to understand and address challenges related to maintaining measles vaccination coverage.

Another relevant issue that hinders results generalization in this review is that the included studies analyzed vaccination in the first year of life along with other age groups. However, different age groups may have distinct determinants that influence both adherence and vaccination coverage levels. For example, factors affecting adult vaccination may differ from those impacting childhood vaccination, and even within childhood, reasons for vaccinating (or not) may vary depending on the child’s age, as observed in studies conducted in Jordan ^10^, Canada ^21^, and Singapore ^39^. This may lead to varying vaccination coverage rates when the results are stratified by age, emphasizing the importance of analyzing these variables separately.

Moreover, regional differences, characteristics of healthcare systems, levels of access, degrees of comprehensiveness, and other contextual factors introduce additional complexities beyond those previously mentioned, leading to unique scenarios that cannot be generalized. Each region has specific socioeconomic, cultural, and political characteristics that shape vaccination dynamics, making it inappropriate to extrapolate results to distinct contexts without careful analysis. Therefore, the heterogeneity among populations and contexts addressed in the studies underscores the need for cautious and context-specific interpretations of the findings in this review.

### Post-block vaccination recovery

In Quebec (Canada), a process was initiated to address gaps in child vaccination, recognizing the vulnerability of children to immunization-preventable diseases. In response, the country has implemented strategies to recover lost or delayed vaccination of unimmunized children, both in Quebec and other Canadian provinces ^21^. Given this context, other authors emphasize the urgent need for detailed records of unvaccinated children, in order to trace them and ensure the administration of vaccine doses that were not administered during periods of interruption ^30^. The studies highlight the need to prioritize not only the recovery of vaccination coverage rates, which declined during critical moments of the COVID-19 pandemic, but also the immunization of the population that did not have access to vaccines during these challenging times. This approach highlights the importance of restoring pre-pandemic vaccination levels and including and re-engaging people who may have been inadvertently excluded from the immunization process. This consideration is key to mitigate immune gaps and prevent outbreaks of vaccine-preventable diseases.

### Intensification of child immunization services in Europe

An investigation carried out to assess vaccination coverage in the Swiss population revealed an increase of approximately 10% in the total coverage of vaccines such as DTaP-IPV-Hib, MMR, and HBV, despite the COVID-19 pandemic ^40^. However, it was found that the complete immunization rate among Swiss children remained below 90%, with several vaccine doses not administered or applied late. This shows that, although vaccination coverage has increased, especially the first dose, the country has still not reached the minimum recommended level. A study conducted in England and Scotland revealed that, in the latter, vaccination coverage with the first dose of MMR increased from 65.2% in 2019 to 78.4% during the first lockdown in the United Kingdom (March 23 to July 26, 2020), while coverage with the second dose of MMR rose from 51.8% in 2019 to 66.1% during the same period ^27^. These results corroborate findings of another study conducted in Switzerland, which indicates an increase in vaccination rates during critical periods of the pandemic ^40^.

Based on these studies ^27^ ^,^ ^40^, it is possible to infer that the pandemic has also had a positive impact on measles vaccination coverage, with an increase observed during lockdowns and mobility restrictions. However, the countries analyzed already had sub-optimal immunization rates before the pandemic, as evidenced by the low measles vaccination coverage rates aforementioned. Therefore, while the temporary vaccination increase during the pandemic is encouraging, there is a clear need to implement ongoing strategies to improve this process and ensure adequate protection against measles and other vaccine-preventable diseases. This scenario reinforces the importance of effective public policies, health education, and easy access to vaccines to achieve and maintain optimum immunization levels among the population.

### Limitations and future research needs

This systematic review has some limitations, such as the relatively small sample of 32 articles, which can limit representativeness and not fully cover global diversity. This may affect generalizability of the results, as certain aspects of the pandemic’s impact on vaccination coverage may not have been fully explored. We recognize the potential publication and selection bias, as studies with more significant results are more likely to be published. This dynamic, combined with the exclusion of gray literature, may have influenced the comprehensiveness of the analyzed evidence, particularly in local or regional contexts. While prioritizing peer-reviewed studies ensured greater rigor and data comparability, the lack of non-indexed epidemiological bulletins may have led to a possible distortion of certain local vaccination coverage dynamics. Furthermore, the diversity of formats, languages, and publication methodologies of these documents could have enriched the analysis with additional perspectives, even though their standardization posed a challenge. The heterogeneity in the quality and consistency of the data presented by the articles was also a significant obstacle. This variability arises from differences in methodological designs, study populations, operational definitions adopted, and how data were collected and reported. Such differences make direct comparisons between studies difficult and may limit the identification of consistent patterns in the results. Furthermore, the lack of uniformity in data presentation, such as the absence of standardized indicators to measure vaccination coverage or report the impacts of the pandemic, can introduce biases and compromise the validity of the analyses. Despite the rigorous methodological approach adopted in this review, these limitations underscore the need for caution when interpreting the findings and highlight the importance of future efforts to standardize data collection and reporting in vaccination studies. Furthermore, even with the end of the COVID-19-related International Public Health Emergency, the effects on measles vaccination coverage may persist over time, requiring continued surveillance and longitudinal studies to assess these changes. Finally, an important limitation of this study is the inclusion criterion adopted, which restricted the analysis to articles published only in English, Spanish, and Portuguese. This approach may have limited the scope and comprehensiveness of the review, considering the wide diversity of languages available in the scientific literature. For this reason, we emphasize the need for future reviews to include more languages and explore more diverse contexts.

Given the limitations of this systematic review and the need to understand the ongoing impact of the COVID-19 pandemic on measles vaccination coverage, several areas emerge for future research. Firstly, longitudinal studies can provide a more detailed analysis of vaccination trends over time, considering different geographical and demographic contexts. Additionally, qualitative research can elucidate the social, cultural, and economic determinants that affect vaccination decisions, helping identify barriers and facilitators. Evaluating the effectiveness of public health interventions and policies implemented during the pandemic is also crucial to increase vaccination coverage and prevent measles outbreaks. Comparative studies between countries and regions can provide valuable lessons on best practices in responding to the pandemic and maintaining immunization programs. Finally, investigating the long-term effects of the pandemic on vaccination coverage and public health is key to inform sustainable and resilient policies. Therefore, investment in research on the impact of the pandemic on measles vaccination is essential to better prevent avoidable diseases and protect the health of the global population.

## Final considerations

The COVID-19 pandemic was an unprecedented global event, triggering wide-ranging impacts across several sectors, including public health and world economy. In the health context, there was significant concern about the possible reduction in vaccination coverage in many countries, resulting from disruptions in health services and mobility restrictions imposed to contain the spread of the virus. However, based on the analysis of the studies included in this systematic review, we identified that the impact of COVID-19 on measles immunization was modest on average, ranging from 1% to 10%. Although some nations have been more affected than others, it is encouraging to see that many countries have managed to recover or come close to the vaccination coverage rates seen before the pandemic. However, it is essential to recognize there is still a substantial gap between vaccination rates currently achieved and targets set internationally. It is therefore imperative to intensify global efforts to strengthen measles immunization programs and ensure that vaccination rates exceed the desired figures. This is crucial not only to prevent outbreaks and epidemics, but also to protect public health and promote the resilience of health systems in the face of future challenges. Continued epidemiological surveillance, investment in health infrastructure, and public education on the importance of vaccination are essential to achieve this goal. In short, while there is a limited impact of COVID-19 on measles immunization, there is still much work to be done to ensure that communities worldwide are adequately protected against this highly contagious disease.
